# Regulatory mechanisms of fluvastatin and lovastatin for the p21 induction in human cervical cancer HeLa cells

**DOI:** 10.1371/journal.pone.0214408

**Published:** 2019-04-02

**Authors:** Chi-Kang Lin, Shu-Ting Liu, Cheng-Chang Chang, Shih-Ming Huang

**Affiliations:** 1 Department of Obstetrics and Gynecology, Tri-Service General Hospital, National Defense Medical Center, Taipei City, Taiwan, Republic of China; 2 Department of Biochemistry, National Defense Medical Center, Taipei City, Taiwan, Republic of China; Marshall University, UNITED STATES

## Abstract

p21, an inhibitor of cyclin-dependent kinase, functions as an oncogene or tumor suppressor depending on the context of a variety of extracellular and intracellular signals. The expression of p21 could be regulated at the transcriptional and/or post-translational levels. The p21 gene is well-known to be regulated in both p53-dependent and -independent manners. However, the detailed regulatory mechanisms of p21 messenger RNA and protein expression via statins remain unknown, and the possible application of statins as anticancer reagents remains to be controversial. Our data showed that the statins—fluvastatin and lovastatin—induced p21 expression as general histone deacetylase inhibitors in a p53-independent manner, which is mediated through various pathways, such as apoptosis, autophagy, cell cycle progression, and DNA damage, to be involved in the function of p21 in HeLa cells. The curative effect repositioning of digoxin, a cardiovascular medication, was combined with fluvastatin and lovastatin, and the results further implied that p21 induction is involved in a p53-dependent and p53-independent manner. Digoxin modified the effects of statins on ATF3, p21, p53, and cyclin D1 expression, while fluvastatin boosted its DNA damage effect and lovastatin impeded its DNA damage effect. Fluvastatin and lovastatin combined with digoxin further support the localization specificity of their interactivity with our subcellular localization data. This study will not only clarify the regulatory mechanisms of p21 induction by statins but will also shed light on the repurposing of widely cardiovascular medications for the treatment of cervical cancer.

## Introduction

p21^WAF1CIP1^ (hereafter labeled p21), an inhibitor of cyclin-dependent kinase, functions as an oncogene or tumor suppressor in response to a variety of extracellular and intracellular signals [1, 2]. As a functional role of oncogene or tumor suppressor, the expression of p21 can either promote or inhibit tumorigenesis, depending on the cellular context. p21 is frequently deregulated in human cancers and its expression could be regulated at the transcriptional and/or post-translational levels, which is frequently dysregulated in various human cancers. The p21 gene is known to be regulated in a p53-dependent or p53-independent manner, including nuclear receptors, Sp1, and Zac1 [3–8]. Several anticancer agents, such as histone deacetylase inhibitors (HDACIs), at least partially function through their ability to induce the p21 expression [9]. Nevertheless, the involved regulative mechanisms of p21 gene expression via statins remain to be investigated.

Statins inhibit the production of endogenous cholesterol via disrupting 3-hydroxy-3-methylglutaryl-CoA reductase (HMGR) and subsequently block protein prenylation to reduce cell proliferation and migration [10, 11]. Dr. Chen’s laboratory identified the direct interaction of the carboxylic acid moiety of statins with the catalytic site of HDAC1/2 by computational modeling and further suggested a novel mechanism, in which statins not only abrogate HDAC activity but promote histone H3 acetylation to regulate p21 expression [12]. Based on the key structural elements of statins, a series of dual-efficacy compounds were further designed on the purpose of blocking both HDAC and HMGR simultaneously. Results revealed that dual-action compounds not only effectively reduce HMGR activity but also promote the acetylation of histone and tubulin in cancer cells [13]. However, the potential of administering statins as anticancer agents still need to be verified.

Drug repositioning, or drug repurposing, has now become a powerful and efficient alternative strategy for the discovery and development of novel anticancer drug candidates. For instance, many widely administrated cardiovascular medications, including cardiac glycosides, statins, and β-blockers, have been demonstrated to possess additional pharmaceutic efficacy targeting cancer therapeutics and prevention [14–16]. In the original scenario, cardiac glycosides such as Digoxin and ouabain that are widely used in the treatment of congestive heart failure and arrhythmia have the pharmaceutic efficacy in blocking the activity of Na^+^/K^+^ ATPase, which resulting in the increase of intracellular calcium ions and subsequently enhancing calcium-dependent signaling and myocardial contractility [17, 18]. Recent studies have shown that digoxin and ouabain reduce the expression level of p53 by activating Src signaling pathways, disrupting p53 protein synthesis via intracellular potassium depletion, or mediating the serine/arginine-rich splicing factor 3 (SRSF3)-dependent alternative splicing of p53 from the α isoform into theβ isoform [15, 19, 20]. Alternatively, cardiac glycosides is an essential factor in apoptosis, immunogenic apoptosis, and non-sense-mediated mRNA degradation of tumor cells via the sustained increased of [Ca^2+^]_i_ [20–24]. However, the detailed mechanisms of cardiac glycoside-induced cell death need to be further investigated.

To date, statins and cardiac glycosides have advanced to clinical trial testing in cancer therapeutics, such as recurrent or metastatic squamous cell carcinoma of the head and neck or of the cervix [14, 25]. It remains unclear whether the promising preclinical activity observed with statins and cardiac glycosides translates into clinically meaningful outcomes. In this study, we aimed to examine the combination of statins with various HDACIs or digoxin in terms of their p21-induction mechanisms in human cervical carcinoma (HeLa) cells. HeLa cells are derived from human papillomavirus (HPV)-infected cervical carcinomas [26]. HPV E6-mediated p53 degradation serves as the major mechanism for inactivating p53 and promoting cervical carcinogenesis due to the rare mutation of p53 gene in cervical cancer [27]. Our data demonstrate that fluvastatin and lovastatin work similarly to some HDACIs of p21 induction in a p53-independent manner, but only fluvastatin synergistically worked with some HDACIs in HeLa cells. The complications of the combination of statins with digoxin are predominated by digoxin and worked in a subcellular localization-dependent manner. Thus, this work will provide a reconsideration of widely used cardiovascular medications, including lipid-lowering and Na^+^/K^+^ ATPase blocking medications, for clinical applications and drug repositioning.

## Materials and methods

### Cell culture and reagents

HeLa cells were cultured in DMEM (Dulbecco’s modified Eagle’s medium) supplemented with 10% FBS (fetal bovine serum) and 1% penicillin-streptomycin (Invitrogen, USA). Digoxin, fluvastatin, LBH589, lovastatin, MS-275, NaB (sodium butyrate), TSA (trichostatin A), SAHA (suberoylanilide hydroxamic acid), and VPA (valproic acid) were purchased from Sigma or Cayman Chemical.

### Western blot analysis

The cell was lysed with RIPA (radioimmunoprecipitation assay) lysis buffer (150 mM NaCl, 0.1% SDS, 100 mM Tris-HCl of pH 8.0, and 1% Triton X-100) and then centrifuged at 12,000 r.p.m., 4°C for 15 min. The supernatant of each sample was mixed with protein loading dye, equally loaded into and subsequently separated by SDS-PAGE, transferred to a polyvinylidine difluoride membrane (Millipore, USA), and finally incubated with primary antibodies against ATF3, p21, p53, cyclin D1, α-actinin (ACTN), histone H3 phosphorylation (at serine 10), HSP90α/β (Santa Cruz Biotechnology, USA), PARP, LC3B (light chain 3B) (Cell Signaling, USA), and γH2A.x (phosphorylated form of H2A.x at serine 139) (Epitomics, USA) at 4°C overnight, followed by the secondary antibody incubation and ECL detection.

### Reverse transcription-polymerase chain reaction (RT-PCR)

One microgram of total RNA, isolated using the TRIsure (BIOLINE, UK) reagent according to the manufacturer’s instructions, was applied and reversely transcript by MMLV reverse transcriptase at 37°C for 1hr (Epicentre Biotechnologies, USA). The PCR amplification reactions were performed on GeneAmp PCR system 9700 (Applied Biosystems, USA). The primer sequences utilized in this study were listed in Table 1.

**Table 1:** PCR primers.

Gene name	Primer sequence (5'→3')
***p53***	Forward: 5'-CTCTGACTGTACCACCATCCACTA-3' Reverse: 5'-GAGTTCCAAGGCCTCATTCAGCTC-3'
***GAPDH***	Forward: 5'-CTTCATTGACCTCAACTAC-3' Reverse: 5'-GCCATCCACAGTCTTCTG-3'
***p21***	Forward: 5'-CTGAGCCGCGACTGTGATGCG-3' Reverse: 5'-GGTCTGCCGCCGTTTTCGACC-3'
***ATF3***	Forward: 5'-GAGGATTTTGCTAACCTGAC-3' Reverse: 5'-TAGCTCTGCAATGTTCCTTC-3'
***cyclin D1***	Forward: 5'-ATGGAACACCAGCTCC-3' Reverse: 5'-TCAGATGTCCACGTCCCGC-3'

### Fluorescence-activated cell sorting (FACS) analysis

For cell cycle phase distribution evaluation, HeLa cells were harvested and washed with ice-cold PBS (phosphate buffered saline), then incubated in the dark with 0.05% propidium iodide (PI) solution (PI in PBS, 0.1% Triton X-100, and 0.01% RNase added) for 15 min at room temperature. The prepared samples were applied to FACS analysis for the measurements of the DNA content of nuclei labeled with PI, and the distribution of cell cycle phase was analyzed by FACS Calibur flow cytometer (BD Biosciences, USA) [28].

### Subcellular protein fractionation

HeLa cells were grown to confluence in 100-mm culture dishes. The cells were harvested with trypsin-EDTA and then centrifuged at 500 x g for 5 min. The cell pellet was washed by suspending the cell pellet with ice-cold PBS and centrifuging at 500 x g for 2–3 min. A pipette was used to carefully remove the supernatant and to leave the cell pellet as dry as possible. The cell pellet was suspended with ice-cold cytoplasmic extraction buffer containing protease inhibitors. Cytoplasmic and nuclear extracts were prepared using the instructions provided for the Subcellular Protein Fractionation Kit for Cultured Cells (Thermo Scientific, USA).

## Results

**Fig 1:**
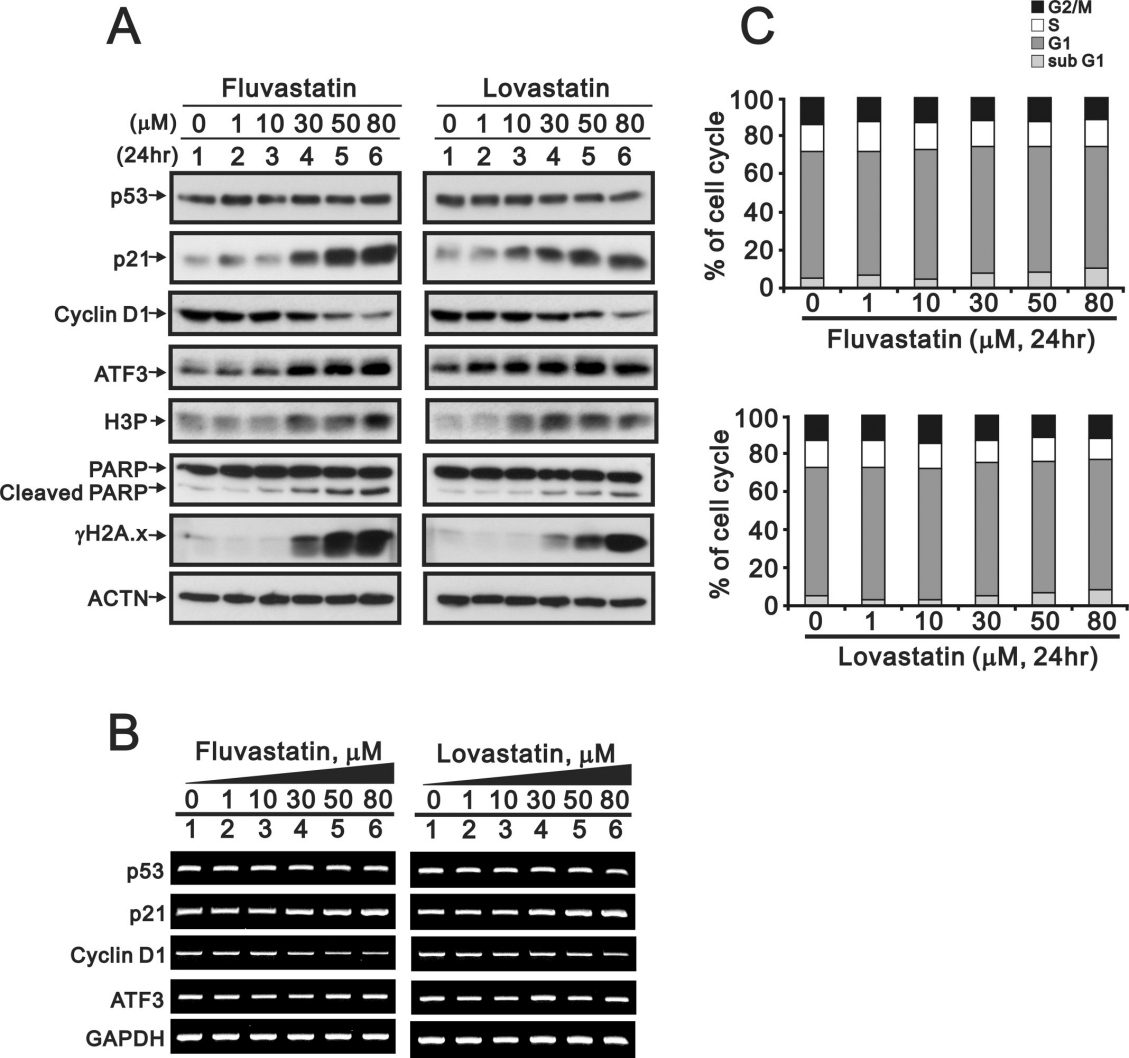
Potential effects of fluvastatin and lovastatin on the target proteins and mRNAs in HeLa cells. The indicated amount of fluvastatin or lovastatin was applied into HeLa cells for 24 h. Then the cells were lysed, collected, and applied to (A) immunoblot analysis for the detection of p53, p21, ATF3, cyclin D1, H3P, PARP (as well as cPARP), γH2A.x and ACTN (loading control), (B) The mRNA expression of p53, p21, cyclin D1, ATF3 and GAPDH (loading control) analyzed by RT-PCR and (C) flow cytometry for cell cycle profile identification. The results are representative of two independent experiments.

Statins have been reported to be inhibitors of histone deacetylase activity to increase p21 expression [12]. We first checked whether p21 proteins are induced by fluvastatin and lovastatin in the HeLa cells (human cervical carcinoma cell line). Our data revealed that p21 induction in HeLa cells is more sensitive to lovastatin than fluvastatin for (Fig 1A and 1B), whereas the p53 levels were constant. Through immunoblotting analyses, the levels of ATF3, H3P (required for entry into mitosis), cleaved PARP (apoptotic effector), and γH2A.x (DNA damage marker), as well as p21 proteins, were increased with an increase in the statin dosage. The abundances of p21 and ATF3 mRNA were marginally increased (Fig 1B). The cyclin D1 protein and gene levels decreased with an increase in the statin dosage (Fig 1A and 1B). The cell cycle profile obtained through flow cytometry analysis demonstrated that a higher dosage of statin increased the percentage of the subG1 population accompanied with a decrease in the percentages of the G1, S, and G2/M populations (Fig 1C).

**Fig 2:**
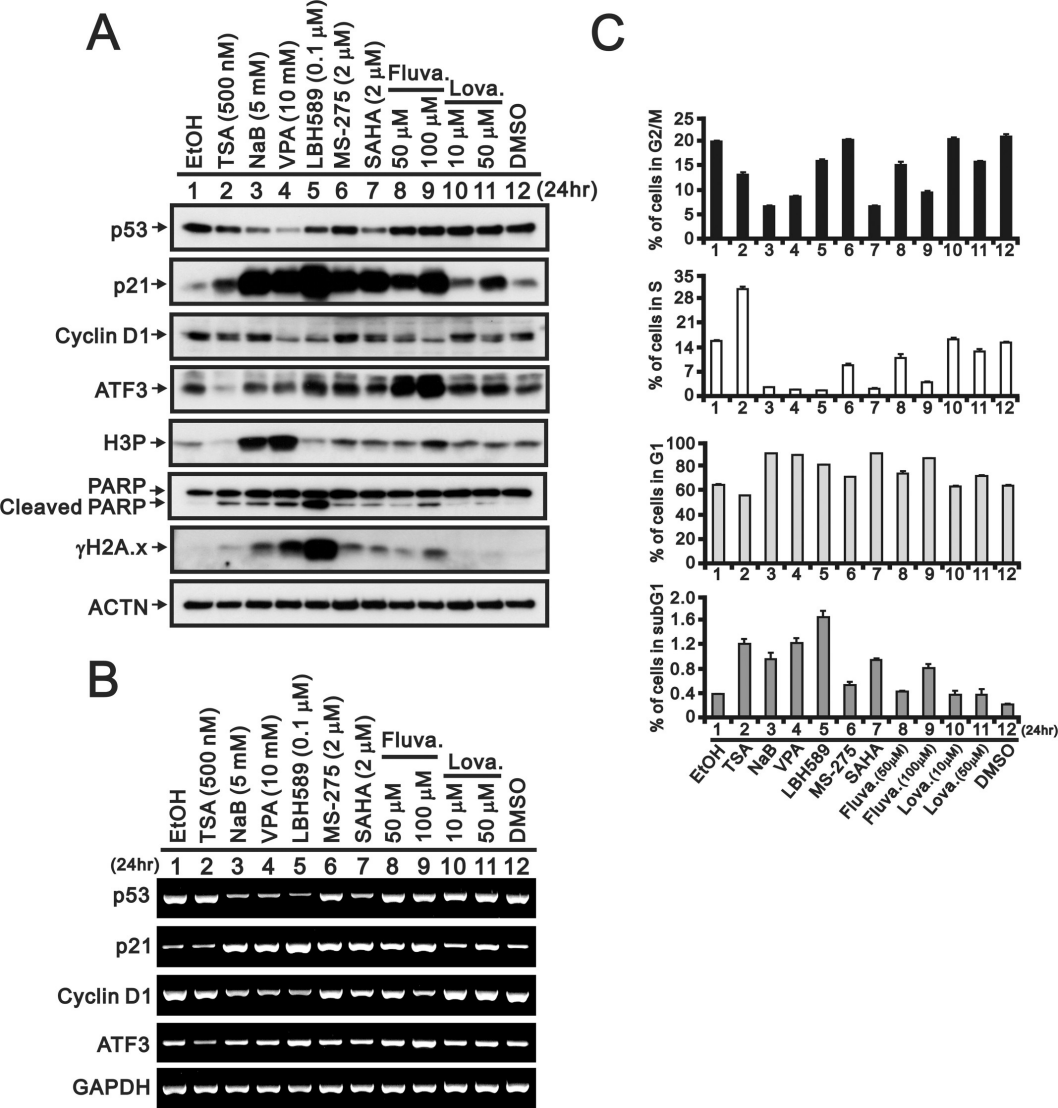
Comparison of fluvastatin and lovastatin with HDACIs in HeLa cells. The indicated amount of the indicated HDACI, fluvastatin or lovastatin was introduced to HeLa cell for 24 h. The HeLa cells were lysed, collected, and applied to (A) western blot analysis for the protein level of p53, p21, cyclin D1, ATF3, H3P, PARP (and cleaved PARP), γH2A.x and ACTN (loading control), (B) RT-PCR analysis of p53, p21, cyclin D1, ATF3 and GAPDH (loading control) expression and (C) flow cytometry for analysis of the cell cycle profile. The results are representative of two independent experiments.

**Fig 3:**
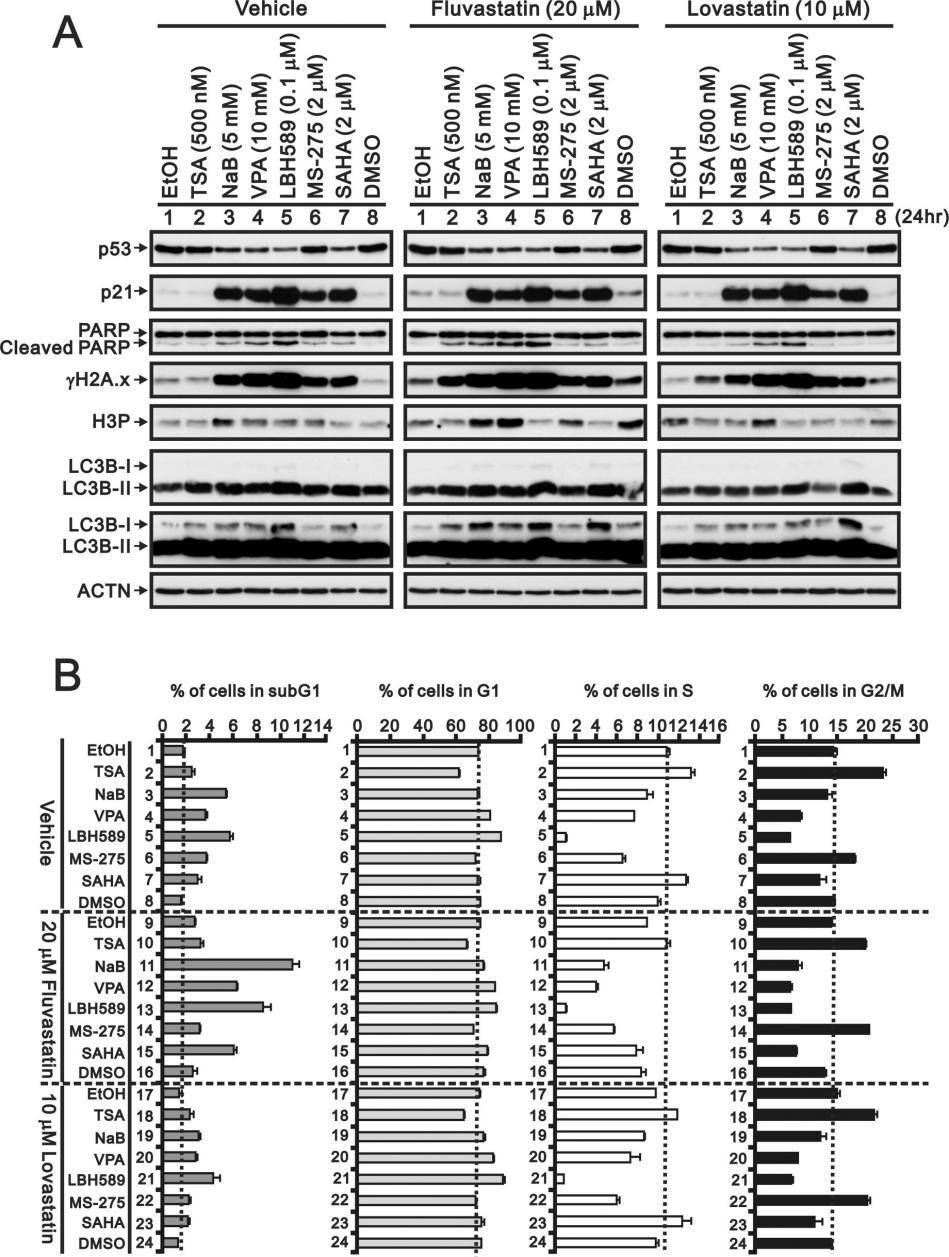
Combinatory effect of fluvastatin and lovastatin with HDACIs in HeLa cells. HeLa cells were treated with the indicated amount of the indicated HDACI and either fluvastatin or lovastatin for 24 h. The cells were lysed, harvested, collected, and applied to (A) western blot analysis for the detection of p53, p21, PARP (and cleaved PARP), γH2A.x, H3P, LC3B and ACTN (loading control) and (B) flow cytometry for analysis of the cell cycle profile. The results are representative of two independent experiments.

We then compared these two statins, namely fluvastatin and lovastatin, with various structural derivatives of the HDACI, including TSA, NaB, VPA, LBH589, MS-275, and SAHA, through western blotting, RT-PCR and flow cytometry analyses (Fig 2). Compared with the well-known HDACI effect on the p53-independent p21 gene and protein expression, fluvastatin and lovastatin as well as the classical HDACI induced p21 gene and protein expression in HeLa cells (Fig 2A and 2B). Positive and negative effects on p53, cyclin D1, and ATF3 (activating transcription factor 3) gene and protein expression, similarly to those obtained with most of the HDACI, were observed. We also examined the abundances of a DNA damage marker γH2A.x (phosphorylated form of H2A.x at serine 139), and an apoptosis marker cleaved PARP and the levels of histone H3 phosphorylation (at serine 10) (Fig 2B). A higher amount of fluvastatin has inductive effects on the abundances of γH2A.x, cleaved PARP (cPARP), and H3P in HeLa cells (Fig 2B). Fluvastatin, but not lovastatin, had similar effects on the cell cycle profile pattern compared with most of the tested HDACIs, as determined through flow cytometry analysis (Fig 2C). However, the TSA effects in HeLa cells, including the ATF3 and H3P levels and the cell cycle profile, were different from those obtained with the other tested HDACIs. We further examined whether the effects of statins were mediated through mechanisms similar to those induced by HDACIs. Fluvastatin had more comparative effects than lovastatin on the target proteins of HDACIs, including p21, cyclin D1, ATF3, γH2A.x, cPARP, H3P, and LC3B (light chain 3B) I/II (Fig 3A). We observed that fluvastatin exerted a positive cumulative effect on the subG1 and G1 populations and a negative cumulative effect on the S and G2/M populations in HeLa cells (Fig 3B). In general, lovastatin had no apparent effect when combined with the HDACIs under our experimental conditions.

**Fig 4:**
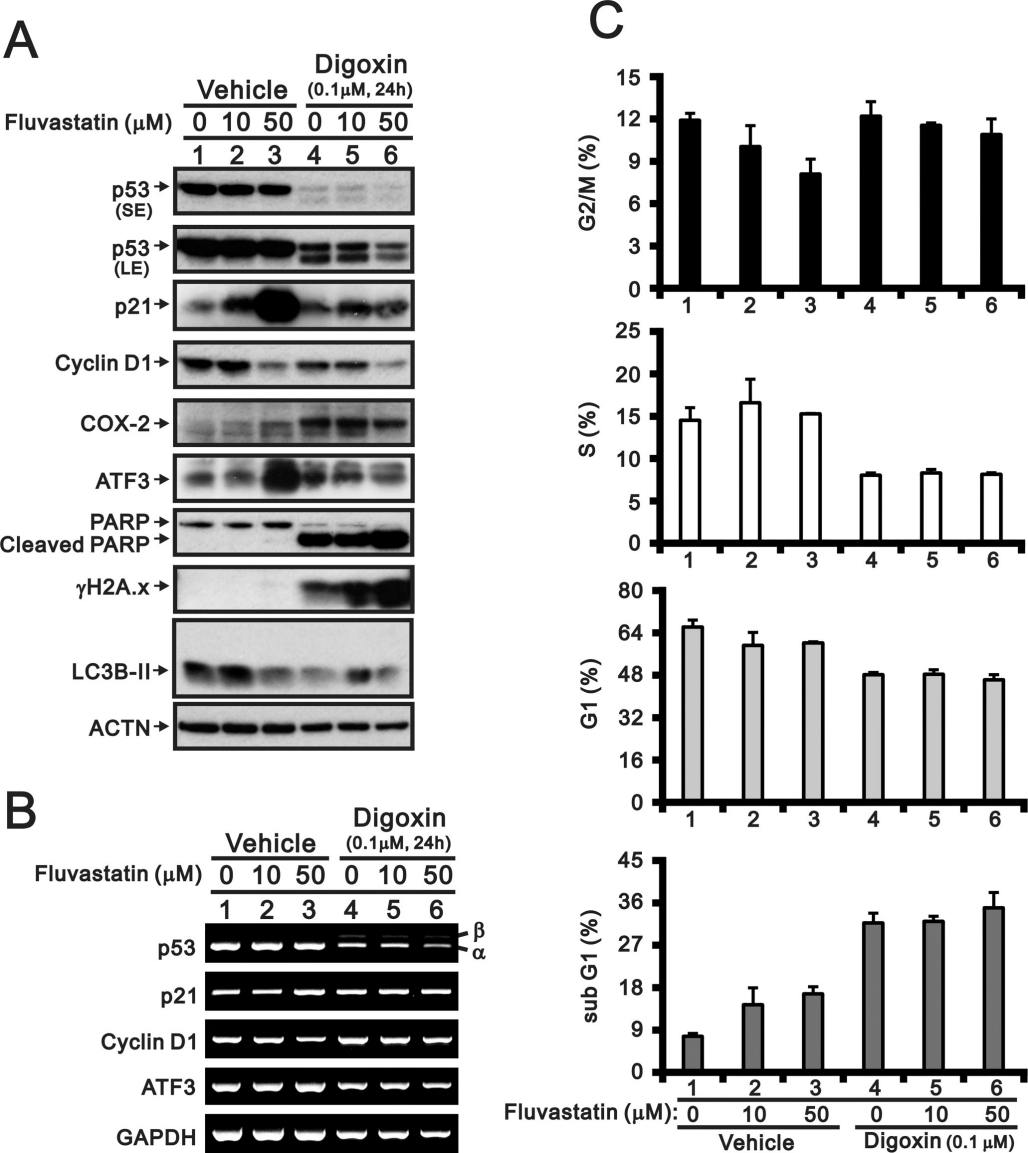
Combinatory effect of fluvastatin with digoxin in HeLa cells. The human cervical carcinoma cells were treated with the indicated amount of fluvastatin with or without 0.1 μM digoxin for 24 h. The cells were lysed, collected, and applied to (A) western blot for the detection of p53 (SE: shorter exposure; LE: longer exposure), p21, cyclin D1, ATF3, Cox-2, PARP (and cleaved PARP), γH2A.x, LC 3B and ACTN (loading control), (B) RT-PCR analysis of p53, p21, cyclin D1, ATF3 and GAPDH (loading control) expression and (C) flow cytometry for analysis of the cell cycle profile. The results are representative of two independent experiments.

**Fig 5:**
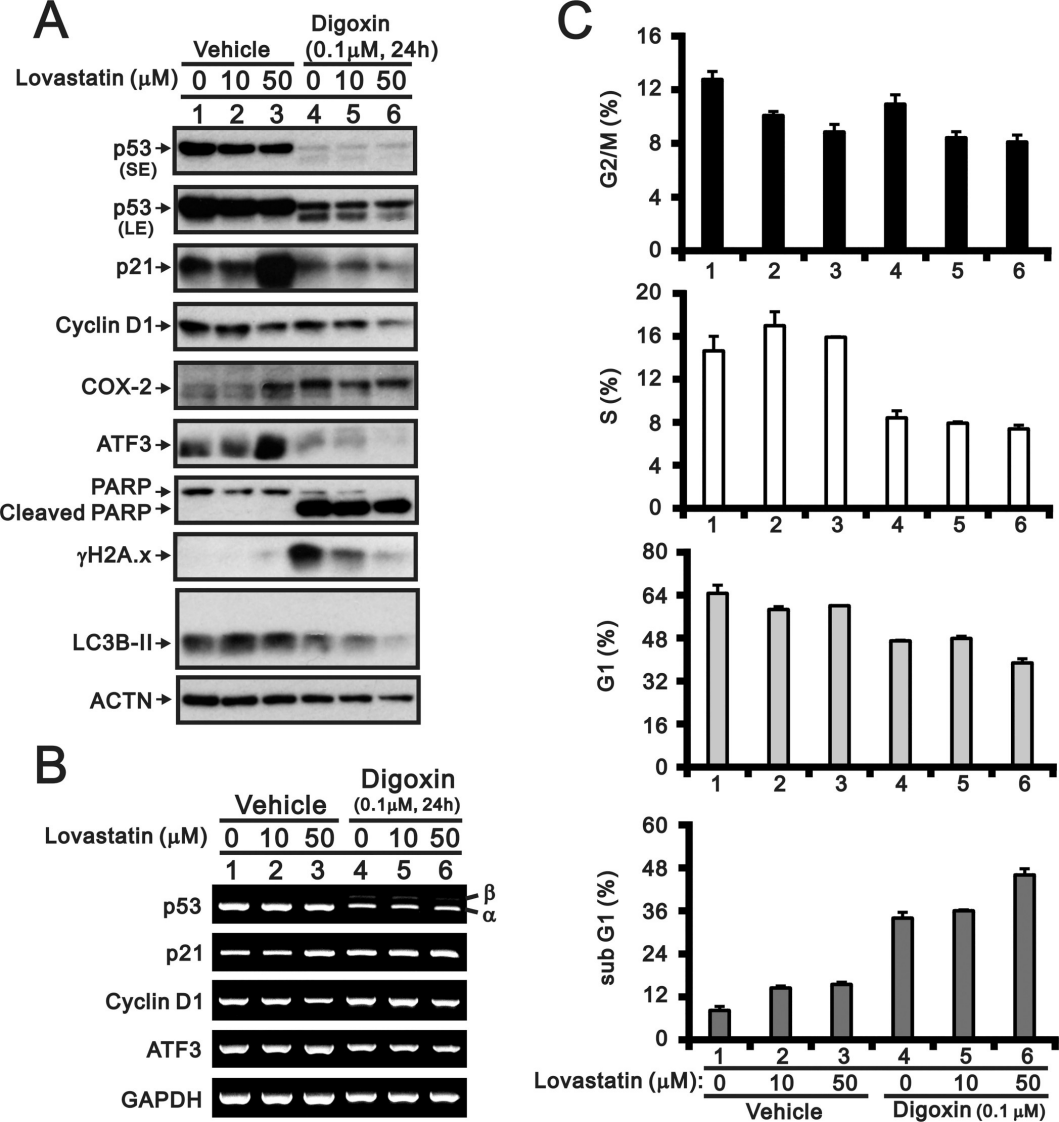
Combinatory effect of lovastatin with digoxin in HeLa cells. HeLa cells were treated with the indicated amount of lovastatin with or without 0.1 μM digoxin for 24 h. The cells were lysed, collected, and subjected to (A) western blot for the detection of p53 (SE: shorter exposure; LE: longer exposure), p21, cyclin D1, ATF3, Cox-2, PARP (and cleaved PARP), γH2A.x, LC3B and ACTN (loading control), (B) RT-PCR analysis of p53, p21, cyclin D1, ATF3 and GAPDH (loading control) expression and (C) flow cytometry for analysis of the cell cycle profile. The results are representative of two independent experiments.

A recent study demonstrated that digoxin may be involved in anti-tumorigenesis through reductions in p53 mRNA and protein level in HeLa cells [15]. In this study, we examined the combinatory effect of fluvastatin or lovastatin with digoxin in HeLa cells (Figs 4 and 5). In addition to the reduction and switch of p53 expression, the inductive effects of fluvastatin on p21 and ATF3 proteins were suppressed by digoxin (Fig 4A). Digoxin enhanced the effects of fluvastatin on Cox-2, γH2A.x, and cPARP and suppressed the effects of fluvastatin on cyclin D1 and LC3B I/II (Fig 4A). Digoxin suppressed the fluvastatin-induced reduction in the p21, cyclin D1, and ATF3 gene expression levels (Fig 4B). The analysis of the cell cycle profile showed that the effects of the fluvastatin dosage on each phase were predominately modulated by digoxin when HeLa cells were co-treated with fluvastatin and digoxin, as determined through FACS analysis (Fig 4C). Although lovastatin had a similar effect to fluvastatin with or without digoxin, as shown in Fig 4A and 4B (Fig 5A and 5B), it resulted in a measurable difference in the γH2A.x abundance.

**Fig 6:**
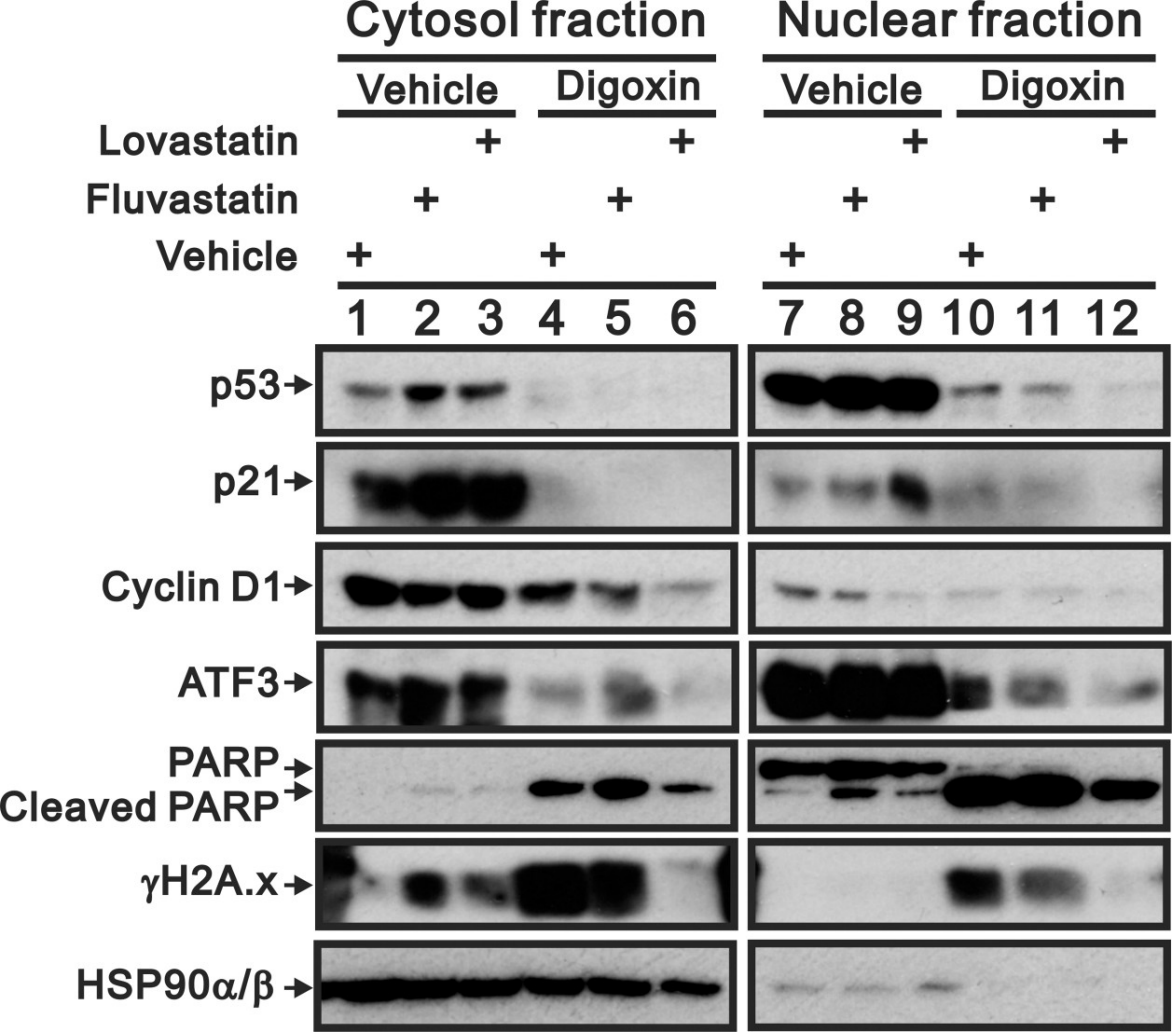
Subcellular localization of the combinatory effect of fluvastatin and lovastatin with digoxin in HeLa cells. HeLa cells were treated with 50 μM fluvastatin or lovastatin with or without 0.1 μM digoxin for 24 h. The cells were fractioned into the cytosol and nuclear fractions and subjected to immunoblot analysis for the detection of p53, p21, cyclin D1, ATF3, PARP (and cleaved PARP), γH2A.x and HSP90α/β (cytosol fraction marker). The results are representative of two independent experiments.

We further fractionated drug-treated HeLa cells and found that fluvastatin and lovastatin affected the p53, p21, cyclin D1, and ATF3 proteins in both the cytosol and nuclear fractions, whereas the effect on γH2A.x was observed in the cytosol fraction, and the effect on cleaved PARP was primarily observed in the nuclear fraction (Fig 6). In addition, digoxin suppressed the effects of fluvastatin and lovastatin on the p53, p21, cyclin D1, and ATF3 proteins in the both cytosol and nuclear fractions. The enhancement of γH2A.x and cPARP abundances by digoxin was found in both the cytosol and nuclear fractions.

## Discussion

In this study, we examined the effect of the combination of statins with six HDACIs (TSA, NaB, VPA, LBH589, MS-275 and SAHA) or digoxin on p21 induction and cell death mechanisms. In addition to human cervical HeLa cells, a similar study had been presented in glioblastoma multiforme cells [16]. Our data first demonstrated that fluvastatin and lovastatin worked similarly to some HDACIs, which were shown in previous studies to induce p21 gene and protein expression [16]. We further demonstrated that only fluvastatin synergistically worked with four of the HDACIs, namely NaB, VPA, LBH589 and SAHA, in HeLa cells. The combination of statin and digoxin demonstrated the predominance of digoxin in most analytical systems, but the γH2A.x induction in HeLa cells could be suppressed by statins, particularly lovastatin. Our findings consistently support that various mechanisms might be involved into the regulation of p21 mRNA and protein in a cell including transcriptional regulation, epigenetic regulation, mRNA stability, and ubiquitin-dependent and ubiquitin-independent degradation of the protein [1, 2]. Hence, our study not only provides candidate working pathways of statins that are similar to those of well-known HDACIs but also demonstrates the complexity of statins combined with cardiac glycosides in anti-cancer therapy.

In this study, we observed some different characteristics between fluvastatin and lovastatin. The possible reasons may be caused by the subtle differences in the HMGR-binding modes and/or targets, such as proteins in the HDAC family, between these two compounds [12, 29]. Statins, as well as HDACIs, induce p21 gene and protein expression via a p53-independent pathway [12, 13]. As we know, the different functions of p21 in regulating genes and its role involved in genomic stability, apoptosis, aging, autophagy and DNA repair might lead to the development of cancers. However, it could have a profound impact on DNA damaging agents or the efficacy of other anti-cancer drugs which will induce activation of p21 [1, 2]. The distinct functions of subcellular localization of p21 proteins: nuclear p21 inhibits cell growth and cytosol p21 induces anti-apoptotic or oncogenic activities. In our case, statin-induced p21 proteins were distributed in both the cytosol and nuclear fractions. The challenges that lie ahead of us are how to inhibit p21 carcinogenic activity selectively, but to maintain its tumor suppressor function simultaneously.

At present, it remains unclear which transcriptional factors are responsible for statin-induced p21 expression. In general, the p21 gene is regulated in a p53-dependent or/and p53-independent manner [3–8]. A recent study showed that TSA induces p21 expression via the down-regulation of ATF3 in A431 epidermoid carcinoma cells [30]. In HeLa cells, we observed similar effects of TSA on p21 and ATF3, whereas the other tested HDACIs induced both p21 and ATF3 expressions. The work conducted by Dr. Yan’s laboratory demonstrated that ATF3 induces p21 expression via the activation of p53 to prevent HPV E6-associated protein from binding to HPV E6 [31], and ATF3 proteins, as well as p53 proteins, are degraded through a p53-dependent mdm2 pathway [32]. In this study, drug-induced ATF3 expression may be mediated through the E6-p53 pathway to increase the p21 abundance in HeLa cells. However, the crosstalk between p21 and the feedback loop of p53/mdm2 (or grail) is complicated [33, 34]. More important, the functional role of ATF3 on p21 gene expression remains to be further investigated.

## Conclusion

Here, our findings suggest that statins fluvastatin and lovastatin might work as general HDACIs to induce p21 expression in a p53-independent manner, selectively cooperates through numerous pathways, such as apoptosis, autophagy, cell cycle progression as well as DNA damage, with various drugs, including HDACIs and digoxin, in HeLa cells. Hence, this study will not only provide the regulatory mechanisms of p21 induction by statins but will also enable the repurposing of widely cardiovascular medications for cervical cancer treatment.

## Supporting information

S1 Table The data of potential effects of fluvastatin and lovastatin in the cell cycle profile. See corresponding plot in Fig 1C.
(XLSX)

S2 Table The data of comparison of fluvastatin and lovastatin with HADC inhibitors in in the cell cycle profile. See corresponding plot in Fig 2C.
(XLSX)

S3 Table The data of combinatory effect of fluvastatin and lovastatin with HADC inhibitors in the cell cycle profile. See corresponding plot in Fig 3B.
(XLSX)

S1 File The data of combinatory effect of fluvastatin and lovastatin with digoxin in the cell cycle profile. See corresponding plots in Figs 4C and 5C.
(XLSX)
